# Noninvasive ventilation as a weaning-facilitating strategy

**DOI:** 10.1186/2197-425X-3-S1-A171

**Published:** 2015-10-01

**Authors:** P Guijo González, JM Estella, A Ramos Rodríguez, M Jaen Franco, M Recuerda, T Rico Armenteros, L Fernández Ruiz, A Jareño Chaumel

**Affiliations:** Hospital del SAS de Jerez, Intensive Care Unit, Jerez de la Frontera, Spain

## Introduction

Extubation of critically ill patients with cardiac dysfunction and/or hypercapnic chronic obstructive pulmonary disease are probably one of the most common causes of weaning failure. Both extubation delay and especially the need for reintubation are associated with poor outcomes and an increase in morbidity.

## Objectives

The aim of the present study was to evaluate the frequency of extubation failure in critically ill patients with chronic obstructive pulmonary disease (COPD) and/or cardiac failure and to analyze the efficacy of non invasive ventilation (NIV) applied inmediately after planned extubation.

## Methods

Observational study performed in a 17 beds medical-surgical ICU. Time of study was 14 months. Consecutive mechanically ventilated patients with COPD and/or cardiac failure were included. The variables analyzed were age, sex, ICU diagnosis, APACHE II at admission in ICU, fluid balance, ICU length of stay, tracheotomy, rate of reintubation. We distinguish two strategies of extubation based in physician´s criteria according with or without the use of NIV after planned extubation.

## Results

44 patients were finally included. 26 were treated with NIV immediately after planned extubation.

Shows clinical characteristics of patients analyzed. Reintubation rate was significantly lower in the NIV after extubation strategy subgroup of patients (p < 0.05, RR, 0.167; 95% CI 0.029-0.953). In our series we observed a higher mortality in extubated patients without use of NIV.

## Conclusions

A high rate of reintubation was observed in mechanically ill patients with COPD and/or cardiac failure.

Preventive use of NIV immediately after planned extubation was associated with a minor reintubation rate. NIV may be considered like an useful tool in patients with high risk of developing respiratory failure after extubation.

**Table 1:** Study characteristics.

	NIV after extubation strategy (n: 26)	Extubation without NIV (n: 18)
Age	68	66
Apache II at admission	22.3	21.1
24 h fluid balance	-1126	-1979
Ventilator Associated Pneumonia (%)	7.7%	11.1%
ARDS (%)	11.5%	5.6%
Tracheotomy (%)	0%	11.1%
ICU lenght of stay	10.4	9.6
Reintubation rate (%)	7.7%	33.3%
Mortality (%)	3.8%	11.1%

